# Antitumor properties of griseofulvin and its toxicity

**DOI:** 10.3389/fphar.2024.1459539

**Published:** 2024-09-03

**Authors:** Nanqiong Yu, Yixiao Fu, Qingkui Fan, Li Lin, Zhifeng Ning, Dongze Leng, Meichun Hu, Tonghui She

**Affiliations:** ^1^ Key Laboratory of Environmental Related Diseases and One Health, School of Basic Medical Sciences, Xianning Medical College, Hubei University of Science and Technology, Xianning, China; ^2^ School of Stomatology and Ophthalmology, Xianning Medical College, Hubei University of Science and Technology, Xianning, China

**Keywords:** griseofulvin, derivatives, antitumor, toxicity, mechanism

## Abstract

Griseofulvin (GF), which is mainly extracted from *Penicillium griseofulvum*, is a heat-resistant, chlorine-containing non-polyene antifungal antibiotic. Previous research shows that GF has a variety of pharmacological effects, such as anti-inflammatory, antifungal, antiviral, and antitumor effects. In recent years, GF has received extensive attention for its antitumor effects as a natural compound, offering a low price, a wide range of uses, and other beneficial characteristics. However, no comprehensive review of GF pharmacological activity in tumors has been published so far. In order to fully elucidate the antitumor activities of GF, this review focuses on the antitumor potential and toxicity of GF and its derivatives, based on a literature search using PubMed, Web of Science, and other databases, to lay a good foundation for further research of GF and the development of new drugs for antitumor activities.

## 1 Introduction

Griseofulvin (GF) is a chlorine-containing metabolite isolated from *Penicillium griseofulvum* by Oxford et al. in 1939. Penicillium is a part of saprophytic fungi, including 97 species such as *Penicillium griseofulvum* and *Penicillium citrinum*. More than 380 metabolites of *Penicillium* have been reported, which are mainly composed of GF, penicillin, citric acid, and cellulase (De Carli and Larizza, 1988; Kong, 2007). Except for *Penicillium*, GF may also be isolated from other ascomycetes, including *Stachybotrys levispora*. GF was originally considered an antibiotic (Ribeiro et al., 2018). In recent years, GF has attracted attention as an anticancer and anti-leukemia drug because it can effectively inhibit the proliferation of different types of cancer cells and has low toxicity (Ho et al., 2001; Uen et al., 2007; Rathinasamy et al., 2010).

Cancer is one of the leading causes of death; due to various factors, it is expected that by 2030, cancer cases will increase by approximately 10.6 million, and cancer deaths will increase by 7.5 million (Kiri and Ryba, 2024; Sankaranarayanan et al., 2014). Cancer is characterized by the uncontrolled growth and reproduction of abnormal cells (Zaimy et al., 2017; Tewari et al., 2022). The occurrence of cancer is a complex process involving multiple factors, including excessive oxidative stress, chronic inflammation, cell cycle disorder, abnormal expression of proto-oncogenes, and angiogenesis dysfunction (Coussens and Werb, 2002; Reuter et al., 2010; Caliri et al., 2021). Chemotherapy is one of the main cancer treatments. However, conventional chemotherapy drugs have the disadvantages of high toxicity, high drug resistance, high cost, and poor applicability (Dong et al., 2024; Greenlee et al., 2000; Pérez-Herrero et al., 2015; Wang et al., 2018). Therefore, it is necessary to find safer, more effective, and cheaper antitumor drugs to enhance the efficacy and improve the prognosis of patients. Natural GF compounds have attracted attention due to their various biological properties, such as inhibition of cell proliferation, cell cycle arrest, and therapeutic potential for a variety of cancers (Ho et al., 2001; Rathinasamy et al., 2010). In addition, the antiviral, anti-inflammatory, antifungal, and malaria prevention effects of GF have also become the focus of research (Gupta et al., 2001; Jin et al., 2008; Tamaki et al., 1995; Chandana et al., 2022).

GF has been widely discussed in previous studies; however, there has been a lack of systematic review. This review focuses on three aspects: (1) the overall characteristics of GF; (2) the potential antitumor effects of GF and its derivatives on different cancers; and (3) the side effects of GF.

## 2 General characteristics of GF

**FIGURE 1:**
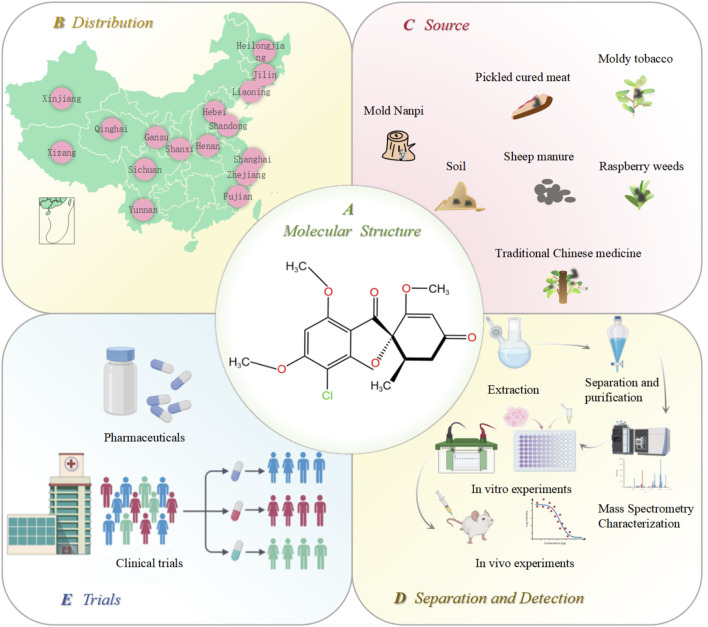
Distribution, source, extraction, and *in vitro* and *in vivo* verification of GF. **(A)** molecular structure of GF; **(B)** distribution of *Penicillium* in various provinces of China; **(C)** source of *Penicillium*; **(D)** GF extraction, separation, purification, and *in vitro* and *in vivo* efficacy and safety verification; and **(E)** GF is finally finalized as a new drug after its effectiveness is confirmed by clinical trials. Figures were created using Biorender.com.

*Penicillium* is one of the most widely distributed and common fungi. It is mainly distributed in Hebei, Liaoning, Jilin, Heilongjiang, Hubei, Xizang, and Xinjiang (Figure 1). Because of its strong resistance to hot, cold, dry, and other adverse environments, it only needs a small amount of humidity to grow. It mainly exists in soil, traditional Chinese medicinal herbs, moldy tobacco, and moldy bacon (Figure 1). The most famous antibiotic, penicillin, is extracted from the species *Penicillium*. Penicillin is the first discovered, first purified, and earliest antibiotic used in clinical practice (Kong, 2007). In 1939, another important antibiotic, GF, was also extracted from *Penicillium griseofulvum*. GF is a natural compound with two methoxy groups at the four and six positions on the aromatic ring structure. It is a white, crystalline powder with a slight bitter taste. Its molecular formula is C_17_H_17_ClO_6_, and the structural formula is shown in Figure 1. Its molecular weight is 352.77 ug, melting point is 218°C–224°C, and boiling point is 570.4°C. It is soluble in ethanol and methanol and insoluble in water. In 1958, GF was first used in clinical practice (Williams et al., 1959; De Carli and Larizza, 1988; Petersen et al., 2014). It has a wide range of pharmacological effects. In agriculture, GF can be used as a plant protective agent to prevent fungal invasion and infection. In medicine, GF is widely used because of its low toxicity, including antifungal (Finkelstein et al., 1996; Ruiz et al., 2020), anti-inflammatory (Wallace and Nisse, 1962), antiviral (Jin et al., 2008; Aris et al., 2022a; Aris et al., 2022b), and antitumor properties (Rathinasamy et al., 2010). The initial application of GF in medical treatment originated from the treatment of guinea pigs with microspores and tinea (Gentles, 1958; Gupta et al., 2001). In addition, GF has a significant effect on non-fungal skin inflammation (such as pigmented purpuric dermatosis), suggesting that it also has certain anti-inflammatory and immunomodulatory effects (Tamaki et al., 1995). Further studies showed that GF could inhibit the replication of the hepatitis C virus. Recent studies have focused on the anticancer activity of GF; however, due to the poor water solubility of GF, its absorption in the intestine is limited, which hinders its *in vivo* research and reduces its druggability to a certain extent. Therefore, how to improve the solubility and bioavailability of GF is the key to improving its efficacy (De Carli and Larizza, 1988). The freeze-drying process, emulsion solvent diffusion method, ball milling method, co-precipitation method, and supercritical carbon dioxide-assisted cyclodextrin complexation method are often used to improve the physical and chemical properties, solubility, and oral bioavailability of GF (Veiga et al., 1998; Trotta et al., 2003; Krull et al., 2015; Wang et al., 2022; Hsiung et al., 2023; Diniz et al., 2023; Ding et al., 2023). When GF was administered subcutaneously in the form of nanoparticles and microparticles, the bioavailability was 60–100%, and the bioavailability after oral administration was 17%. Therefore, subcutaneous injection can be used as a more ideal route of administration for GF (Sigfridsson et al., 2019).

## 3 Antitumor effect of GF and its derivatives

**FIGURE 2:**
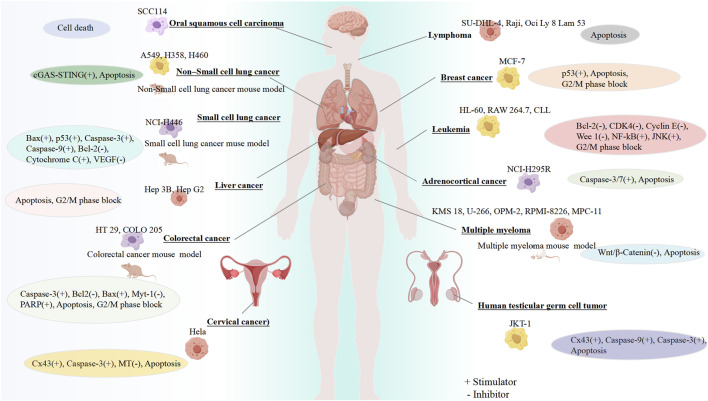
Anticancer properties of GF. Created using FigDraw (www.figdraw.com).

The therapeutic potential of GF for different cancers has been confirmed by a large number of preclinical experiments, highlighting its role in regulating different cancer effects such as inhibiting cell proliferation, blocking the cell cycle, and promoting apoptosis (Figure 2).

### 3.1 Inhibiting the proliferation of tumor cells

At present, many anticancer drugs exert antitumor effects by inhibiting tumor proliferation. Studies have shown that GF inhibits the proliferation of various tumor cells by affecting the function of spindle microtubules in mitosis, including lung, colorectal, breast, cervical, and liver cancer (Ho et al., 2001; Rathinasamy et al., 2010; Panda et al., 2005; Shi, 2012).

It was found that 20 μM GF could significantly inhibit the proliferation of colorectal cancer cell lines (HT-29 and COLO-205), liver cancer cells (Hep 3B and Hep G2), leukemia cells (HL-60), and human normal epidermal keratinocytes (#76 KhGH) for 24 h but had no effect on the proliferation of #76 KhGH cells (Ho et al., 2001). Another report also showed that GF had a significant inhibitory effect on the proliferation of HT-29 and HL-60 cells, and its anti-colorectal cancer properties were further confirmed *in vivo* by establishing a COLO-205 thymus-less mouse model, of which the results showed that GF (50 mg/kg) or ND (5 mg/kg) alone or in combination could effectively inhibit the growth of tumors in nude mice, and the combined effect was better (Uen et al., 2007). While studying the effect of GF on human breast cancer cells (MCF-7), Rathinasamy et al. found that GF had a significant inhibitory effect on MCF-7 cells. When the concentration of GF was 30 μM and 60 μM, the inhibitory effect on MCF-7 proliferation was 73% and 91%, respectively, and the IC_50_ value was 17 ± 2 μM. The mechanism may be related to inhibiting the dynamic changes of microtubules in MCF-7 and activating the tumor suppressor protein p53. In addition, the combination of GF and vincristine had a synergistic effect. When GF and vincristine were used in combination at 10 μM and 0.5 nM or 1 nM, the inhibitory effects on MCF-7 proliferation were 84% and 92%, respectively. Therefore, in future development, GF may be combined with other antitumor drugs to improve the effect of breast cancer treatment (Rathinasamy et al., 2010). In *in vitro* experiments, Schmeel et al. found that GF could significantly inhibit the proliferation of human myeloma cell lines (KMS 18, U-266, OPM-2, and RPMI-8226), the mouse myeloma cell line (MPC-11), and human lymphoma cell lines (SU-DHL-4, Raji, and OCI-Ly 8 Lam 53) in a concentration-dependent manner. The IC_50_ values were 9, 18, 45, 26, 44, 22, 33, and 30 μM, respectively (Schmeel et al., 2017). In general, 10 μM GF can significantly reduce the activity of myeloma cells but only at the cellular level. Therefore, Kim et al. established a mouse myeloma model by inoculating MPC-11 cells and found that GF could significantly inhibit the growth of mouse tumors and prolong the overall survival time, confirming its effectiveness *in vivo* (Table 1). *In vitro* and *in vivo* studies have shown that GF can inhibit the proliferation of myeloma cells and significantly reduce the growth of myeloma by activating the Wnt pathway and reducing the phosphorylation of the β-catenin pathway (Kim et al., 2011). Bramann et al. (2013) used the WST-1 method and [^3^H]-thymidine assay to observe the toxic effect of GF on adrenocortical carcinoma cells (NCI-H295R) and found that GF could inhibit the proliferation of NCI-H295R cells in a dose-dependent manner. Panda et al. (2005) reported that GF could inhibit the proliferation of cervical cancer cells (HeLa) in a concentration-dependent manner, with an IC_50_ value of 20 μM. Sun et al. (2019) also found that the proliferation rate of HeLa cells decreased with the increase in the GF dose; in particular, the proliferation rate of 50 μM and 100 μM dose cells decreased significantly.

**TABLE 1:** Anticancer effects of GF *in vivo*.

Cancer type	Animals	Model	GF concentration	Route of administration	Administration time	Effect	Reference
Non-small-cell lung cancer	4–5 week-old-female and male nude mice	H358 xenograft mouse model	50 mg/kg	Intraperitoneal injection	21 days	Slowed tumor growth	Wang et al. (2023)
Multiple myeloma	BALB/c mice	MCP11 xenograft mouse model	450 μg/day	Oral	60 days	Slowed tumor growth, induced tumor apoptosis, and ↓Wnt/β-catenin	Kim et al. (2011)
Colorectal cancer	Nude mouse	COLO-205 xenograft mouse model	50 mg/kg	Intraperitoneal injection	6 weeks	Inhibitory tumor volume	Ho et al. (2001)
Small-cell lung cancer	4–5 week-old BALB/c-nude male mice	NCI-H446 xenograft mouse model	30 μM/each mouse	Intraperitoneal injection	2 weeks	Slowed tumor growth, ↑Bax, ↑caspase-3,↑p53, ↓Bcl-2, and↓VEGF	Shi (2012)

Furthermore, Mauro et al. found that GF could inhibit the proliferation of the human testicular germ cell tumor cell line (JKT-1) in a concentration-dependent manner after GF was applied to the human testicular germ cell tumor cell line (JKT-1) for 24, 48, and 72 h, with an IC_50_ value of 53 ± 1.7 μM (Table 2) (Mauro et al., 2013). Wang et al. (2023) treated non-small-cell lung cancer cells (A549, H358, and H460) with different concentrations (10–30 μM) of GF and found that 10 μM GF could inhibit the proliferation and colony formation of A549, H358, and H460 cells. Shi et al. found that GF could inhibit the proliferation of small-cell lung cancer cells (NCI-H446) through *in vitro* experiments. Additionally, by further establishing a nude mouse xenograft model *in vivo*, it was found that GF could upregulate the expression of Bax, p53, caspase-3, and other cytokines and downregulate the expression of Bcl-2 and VEGF to inhibit the growth of small-cell lung cancer cells (Shi, 2012). With different concentrations of GF (0–100 μM) applied to oral squamous cell carcinoma (SCC114) cells for 24 h, the proliferation of SCC114 cells was inhibited, with an IC_50_ value of 35 μM (Rebacz et al., 2007). Liéby et al. also confirmed this point in their study, which showed that GF had strong anti-proliferative activity against SCC114 with an IC_50_ value of 6.4 μM. However, whether GF can inhibit the proliferation of other oral squamous cell carcinoma cells remains to be further explored (Liéby-Muller et al., 2015).

**TABLE 2:** Anticancer effects of GF *in vitro*.

Cancer type	Cell lines	GF concentration	IC_50_	Exposure time	Effect	Reference
Breast cancer	MCF-7	15–90 μM	17 ± 2 μM	24 and 48 h	↑p53 and ↓G2/M phase	Rathinasamy et al. (2010)
Multiple myeloma	KMS 18, OPM-2, RPMI-8226, U-266, and MPC-11	0.1–200 μM	KMS 18: 9 μM OPM-2: 45 μM RPMI-822: 26 μM U-266: 18 μM MPC-11: 44 μM	72 h	↓Wnt/β-catenin	Schmeel et al. (2017) and Kim et al. (2011)
Lymphoma	Raji, SU-DHL-4, and Oci Ly 8 Lam 53	0.1–200 μM	Raji: 33 μM SU-DHL-4: 22 μM Oci Ly 8 Lam 53: 30 μM	72 h	—	Schmeel et al. (2017)
Colorectal cancer	HT-29 COLO-205	0–50 μM	—	24 h	↓G2/M phase, ↑caspase-3, ↑PARP, ↑Bax, ↓Bcl-2, and ↓Myt-1	Ho et al. (2001)
Human testicular germ cell tumor	JKT-1	0–100 μM	53 ± 1.7 μM	24, 48, and 72 h	↑Caspase-9, ↑caspase-3, and ↑Cx43	Mauro et al. (2013)
Cervical cancer	Hela	0–120 μM	20 μM	24, 40, 48, and 72 h	↓MT, ↑Cx43, and ↑caspase-3	Panda et al. (2005) and Sun et al. (2019)
Adrenocortical cancer	NCI-H295R	100 nM–100 μM	—	24 and 48 h	↑Caspase-3 and ↑caspase-7	Bramann et al. (2013)
Leukemia	HL-60, RAW 264.7, and CLL	0.1–200 μM	RAW 264.7: 28 μM CLL: 80 μM	24 h 72 h	↑NF-ĸB, ↑JNK, ↓Bcl-2, ↓Wee 1, ↓cyclin E, ↓CDK4, and ↓G2/M phase	Uen et al. (2007), Schmeel et al. (2017), and Ho et al. (2001)
Non-small-cell lung cancer	A549, H358, and H460	0–30 μM	—	48 h	↑cGAS-STING	Wang et al. (2023)
Oral squamous cell carcinoma	SCC114	-	6.4 μM	—	—	Liéby-Muller et al. (2015) and Rebacz et al. (2007)
Small-cell lung cancer	NCI-H446	0–50 μM	24.58 ± 1.32 μM	48 h	↑Caspase-3, ↓Bcl-2, ↑Bax, ↓VEGF, ↑p53, ↑caspase-9, ↑cytochrome C, and ↓G2/M phase	Shi (2012)
Liver cancer	Hep 3B and Hep G2	0–50 μM	—	24 h	↓G2/M phase	Ho et al. (2001)

To verify the antitumor effect of GF derivatives, in *in vitro* experiments, Raab et al. used GF derivatives (GF-15) to act on pancreatic cancer cells (PANC1 and PACA1), cervical cancer cells (HeLa), glioblastoma cells (LN229), colorectal cancer cells (HT-29, HCT116, and SW480), leukemia cells (Ku812, HEL, and MOLM14), primary myeloma cells, and all human multiple myeloma cell lines (RPMI-8226, Dox40, KMS-12BM, NCI-H929, OPM-1, and OPM-2). Studies have shown that GF-15 has a concentration-dependent inhibitory effect on a variety of tumor cells. The average inhibitory concentration IC_50_ was in the range of 1–5 μM. Among them, multiple myeloma and leukemia cell lines are particularly sensitive to the cytotoxicity and anti-proliferative effects of GF-15. The sensitivity of GF-15 to myeloma cell lines is higher than that of Adriamycin and dexamethasone. The mechanism may be related to the dynamic instability of microtubules, abnormal separation or connection of chromosomes, and apoptosis of tumor cells. In the subsequent *in vivo* experiments, an allogeneic tumor model was established by inoculating myeloma OPM-2 cells and colon cancer HT-29 cells for further in-depth analysis. The results showed that GF-15 could significantly inhibit tumor growth and prolong survival (Raab et al., 2012).

To increase the antitumor properties of GF, Liéby et al. synthesized several 20-oxygen and 20-sulfur analogs of GF and studied the antitumor proliferation activity of the synthesized compounds on triple-negative breast cancer (HCC1937) and oral squamous cell carcinoma (SCC114). The IC_50_ values of compounds 4b, 5a, 5b, 5c, 5d, 10b, and 11b against SCC114 were 1.45, 0.4, 2.8, 1, 1.9, 0.25, and 0.4 μM, respectively. Among them, 10a had the highest activity, with an IC_50_ value of only 20 nM. The IC_50_ values of compounds 5a, 5b, 5c, 10b, 11a, and 11b against HCC1937 were 1.4, 5.9, 1.6, 0.4, 0.9, and 0.7 μM, respectively. The IC_50_ value of GF-15 on SCC114 cells was 0.98 μM, which was 27 times higher than that of GF itself (Liéby-Muller et al., 2015). Compared to GF, the 2′-substituted derivatives of GF exhibit a stronger inhibitory effect on microtubule formation. Rebacz et al. synthesized different 2'-substituted GF derivatives to act on SCC114 cells, and their efficacy is about 10 times that of GF itself (Rebacz et al., 2007). Rønnest et al. (2012) modified the 4, 5, 6, 2′, 3′ and 4′ positions of GF, among which 2'-benzyloxy-2'-demethoxy-griseofulvin is one of the most effective compounds against MDA-MB-231 cancer cells. In another study, two new GF derivatives, namely, bostrycin (5) and deoxybostrycin (6), were detected by MTT assay; the results showed that bostrycin (5) had a significant inhibitory effect on the proliferation of MCF-7, Hep G2, A549, KB, Hep-2, and MCF-7/Adr, with IC_50_ values of 6.13, 5.90, 2.64, 4.19, 5.39, and 6.68 μg·mL^-1^, respectively. Deoxybostrycin (6) also inhibited the proliferation of all tumor cell lines *in vitro*, with IC_50_ values of 4.76, 4.41, 2.44, 3.15, 3.15, and 5.46 μg·mL^−1^, respectively (Xia et al., 2011). Teng et al., (2023) obtained three GF derivatives, in which the IC_50_ value of pochonichlamydin C on human breast cancer cell MCF-7 was 33.1 μM.

### 3.2 Inhibition of tumor cell metastasis and invasion

Tumor cells enter the blood or lymphatic system through their adhesion, invasion, and migration ability. The invasion and migration of tumor cells are important steps in their malignant process. Therefore, inhibiting the adhesion, invasion, and metastasis of malignant tumor cells has become the key to the treatment of tumors (Tay et al., 2019). The invasive ability of malignant tumors can be obtained in the early stage. With the deterioration of the disease, the invasive ability of some tumors will gradually increase. The process of adhesion and invasion enhances the proliferation and metastasis of tumors (Jee et al., 1998). Matrigel is a matrix component extracted from mouse Engelbreth–Holm–Swarm (EHS) sarcoma. It contains laminin (LN), type-IV collagen, etc., and can be reconstituted in a culture medium to form a membrane structure, which is very similar to the natural matrix membrane structure and can be used to study the invasion, metastasis, and adhesion of tumor cells (Zheng and Liu, 2003). Shi et al. found using the transwell chamber method and scratch test that when the concentration of GF was 5, 10, and 20 μM, the invasion inhibition rate of NCI-H446 cells was 22.91%, 60.61%, and 100%, respectively, indicating that GF could significantly inhibit the invasion ability of cells at high concentration. The inhibition rates of GF on the longitudinal migration of NCI-H446 cells were 21.82%, 63.42%, and 100% at 5, 10, and 20 μM, respectively, indicating that GF could significantly inhibit the longitudinal migration of cells at high concentrations. The inhibition rates of NCI-H446 cell adhesion ability were 33.32% ± 6.02%, 50.50% ± 4.47%, and 73.62% ± 5.75% when the concentration of GF was 5, 10, and 20 μM, respectively. The inhibition rate increased with an increase in the GF concentration. The above experimental results show that GF has a good inhibitory effect on the migration, invasion, and adhesion of tumor cells (Shi, 2012).

### 3.3 Cell cycle arrest

The cell cycle is an essential part of cell proliferation. Blocking the cell division cycle of tumor cells is one of the important antitumor mechanisms. The cell cycle can be divided according to the characteristics of different periods: G1 phase (pre-synthesis)—this phase involves the synthesis of mRNA, rRNA, tRNA, and nucleosomes for DNA preparation; S phase (DNA synthesis stage)—this is a period of DNA replication during which cells change from diploid to tetraploid; during this period, the synthesis of RNA and protein is active, which prepares the cells to enter the mitotic phase; G2 phase (late stage of DNA synthesis)—RNA and protein synthesis is active in the G2 phase, preparing cells for mitosis; and M phase (cell division stage)—this period is the period from the beginning of cell division to the end (Hinchcliffe and Sluder, 2001; Wang, 2022).

HT-29 cells were treated with 20 μM GF, 0.25 μM nocodazole (ND), and 20 μM etoposide (EP) alone for 24 h. The G2/M phase arrest rates were 41%, 38.8%, and 9.8%. The combination of GF and ND increased the G2/M phase arrest rate from 41% to 95.8%, suggesting that GF combined with ND can significantly promote cell cycle arrest. The mechanism may be related to the increase in cyclin B1 and Cdc2 kinase activity and the decrease in Myt-1 protein expression by regulating the formation of spindle during mitosis (Ho et al., 2001). In MCF-7 cells, GF inhibited the cell cycle progression of MCF-7 cells in the G2/M phase in a dose-dependent manner (Rathinasamy et al., 2010). Uen et al. (2007) found that GF could induce G2/M phase arrest in HL-60 cells by regulating the expression of Wee 1, cyclin E, CDK 4, and other proteins.

Most of the studies published so far have shown that GF treatment induces G2/M cell cycle arrest in tumor cells by regulating the expression of cyclin-regulated proteins, including cyclin B1, cyclin E, and CDK 4 (Ho et al., 2001; Uen et al., 2007; Rathinasamy et al., 2010).

### 3.4 Inducing apoptosis

Apoptosis is the programmed cell death regulated by genes and plays an important role in the development and normal physiological state. Apoptosis involves two major pathways, namely, an endogenous apoptotic pathway guided by mitochondrial/intracellular signals and an exogenous apoptotic pathway guided by death receptors (Newton et al., 2024; Yuan and Ofengeim, 2024). Many experimental studies have shown that GF can induce the apoptosis of a variety of tumor cells.

The results showed that GF induced the apoptosis of colorectal cancer cells by increasing the expression of caspase-3, PARP, and Bax and decreasing the expression of Bcl-2 protein in colorectal cancer cells (Ho et al., 2001). Rathinasamy et al. conducted docking and experimental results through AutoDock4 and LigandFit modules. The results showed that GF could bind to tubulin through two different sites. One site overlapped with the binding site of paclitaxel, and the other site was located in the interface of the αβ dimer. GF formed complexes at two different sites, inhibiting the dynamic movement of microtubules and driving breast cancer cells to undergo programmed death (Rathinasamy et al., 2010). In addition, GF could induce apoptosis by inhibiting the Wnt/β-catenin signaling pathway (KMS 18, U-266, OPM-2, RPMI-8226, and MPC-11) (Schmeel et al., 2017). When NCI-H295R was exposed to GF for 24 h, the cell morphology changed significantly at 10 μM. At 40 μM, obvious apoptosis occurred, and the cell structure basically disappeared. At 100 μM, a large number of apoptotic bodies were observed, with only a small number of living cells. The mechanism may be to induce apoptosis by enhancing caspase-3/caspase-7 activity. However, this study did not compare the effects of GF on normal adrenal cortical cells. Therefore, it is necessary to conduct more in-depth research to evaluate its specific role (Bramann et al., 2013). GF has similar antitumor activity to anti-mitotic drugs such as vinblastine and paclitaxel. Its mechanism of action is to inhibit the polymerization of microtubules, interfere with the formation of spindle microtubules, inhibit cell mitosis, and terminate mitosis, leading to the apoptosis of cervical cancer cells. Considering that GF has no obvious toxic and side effects on the human body, GF can be combined with other more effective drugs in the treatment of cervical cancer (Panda et al., 2005).

Connexin43 (Cx43) is a tumor suppressor gene that is lowly expressed in cervical cancer and is closely related to tumor invasion and metastasis (Steinhoff et al., 2006; Cheng et al., 2015; Rodríguez-Sinovas et al., 2007). The results showed that the expression of the Cx43 protein and mRNA and caspase-3 was increased in HeLa cells induced by GF in a concentration-time-effect relationship, suggesting that GF may induce apoptosis by inducing the high expression of Cx43 in mitochondria and activating caspase-3 activity (Sun et al., 2019). The mechanism study of GF on human testicular germ cell tumor cells showed that GF could induce the apoptosis of JKT-1 cells by activating the expression of Cx43, caspase-9, and caspase-3 (Mauro et al., 2013). In lymphoma, GF may induce apoptosis by inducing a decrease in the mitochondrial membrane potential (Schmeel et al., 2017).

To further understand the effect of GF on non-small-cell lung cancer, a xenograft mouse model was established *in vivo*. The experimental results showed that GF alone or in combination with radiotherapy could induce the production of the micronucleus (MN) *in vitro* and *in vivo* and activate the cyclic GMP-AMP synthase (cGAS) in non-small-cell lung cancer cells. The cGAS is an accelerator of the immune system, from which signals are transmitted to the nucleus through the string pathway, opening the immune response, thereby further inhibiting tumor growth and diffusion, inducing apoptosis, and not affecting normal cells (Wang et al., 2023). Transmission electron microscopy showed that NCI-H446 cells in the GF (20.0 μM) group showed morphological changes in apoptotic cells. The cell morphology was irregular, the cell membrane was basically intact, the cytoplasm was concentrated, the nuclear chromatin was highly condensed and fragmented, and the cytoplasm was vacuolated. Annexin V-FITC/PI double-staining flow cytometry was used to detect GF-induced apoptosis of NCI-H446 cells. After NCI-H446 cells were treated with 10.0 μM and 20.0 μM MGF for 48 h, the apoptosis rates of NCI-H446 cells were 21.65% ± 3.89% and 38.26% ± 3.41%, respectively. The results showed that GF could induce the apoptosis of NCI-H446 cells, and the apoptosis rate increased with an increase in the GF concentration. GF can activate the expression of caspase-3, caspase-9, Bax, and cytochrome C and inhibit the expression of Bcl-2 in NCI-H446 cells, suggesting that GF-induced apoptosis of tumor cells is related to the activation of the mitochondrial cytochrome-C pathway (Shi., 2012). Different concentrations of GF (0–100 μM) acted on SCC114 cells for 24 h, and it was found that GF could induce apoptosis (Rebacz et al., 2007). In leukemia cells (HL-60), GF may induce apoptosis through the NF-kB and JNK pathways (Uen et al., 2007). At the same time, Schmeel et al. observed that GF can also significantly induce apoptosis in mouse leukemia (RAW 264.7) cell lines and primary chronic lymphocytic leukemia (CLL) cells (Schmeel et al., 2017).

## 4 Toxicity of GF

When evaluating the efficacy of a drug, it is crucial to first evaluate its safety and toxicity, and these aspects should be given priority. In the past few decades, many studies have been conducted to evaluate the safety and toxicity of GF.

In cell and animal experiments, it was found that GF had no toxic effect on human normal epidermal keratinocytes (#76 KhGH), and it was further established in a thymus-free mouse model of COLO-205 *in vivo*. After 6 weeks of intraperitoneal injection, it was found that GF had no significant toxic or side effects in nude mice after body weight, overall appearance, and microscopic observations (Ho et al., 2001). Kim et al. established a mouse myeloma model by inoculating MPC-11 cells and found that GF could significantly prolong the overall survival of mice, and no toxic and side effects were observed at the dose applied (Kim et al., 2011). Shi also found that no death of nude mice and other toxic reactions of the body were found during animal experiments (Shi., 2012). It was also reported that GF derivatives had no effect on the cell viability of peripheral blood mononuclear cells (PBMCs) and primary bone marrow stromal cells (BMSCs) (Raab et al., 2012). In a study, different concentrations of GF were used to treat normal fibroblasts (BJ) and human epidermal keratinocytes (NHEK). The results showed that GF had no obvious toxicity (Rebacz et al., 2007). Lastnick also showed that GF is effective, safe, and causes fewer side effects (Lastnick, 1974). In another study, rats were orally administered with 2,000 mg/kg GF per day for 12 weeks. Through immunohistochemistry and histopathological evaluation, it was found that GF was negative in the peripheral blood micronucleus test of rats, and the conclusion was drawn that GF did not initiate the carcinogenic process (Labay et al., 2001). Xu et al. (2012) found that the use of GF can cause side effects such as anorexia, vomiting, and diarrhea after 15 days of GF in dogs with fungal skin diseases. The above results suggest that GF does not cause or only causes mild side effects.

On the contrary, Epstein et al. (1967) reported that GF can induce liver cancer in young mice. Another study also found that mice developed nodular hyperplasia and liver cancer after the administration of GF 12–14 months in mice and athymic nude mice (Chlumská and Janousek, 1981). Rustia et al. found that dietary exposure to GF for 5 weeks could lead to a significant occurrence of hepatocellular tumors in mice and thyroid tumors in rats but had no carcinogenic activity in hamsters (Rustia and Shubik, 1978). Since then, a great number of experiments on the oral administration of high-dose microparticle griseofulvin in mice have been done. The experimental results show that the toxic reactions caused by GF are mainly manifested in porphyrin metabolism disorder and carcinogenicity. However, the toxicity study of GF was successfully carried out in mice at very high doses. No pathological changes were observed in other experimental animals, such as guinea pigs, rats, rabbits, cats, and dogs, at very high doses corresponding to body weight. Since the widespread application of GF in the treatment of cutaneous fungal diseases, there has been no clinical report of severe poisoning or tumor changes (1973).

Clinically, drug-induced liver injury and serious adverse reactions are one of the main reasons for the failure of drug clinical trials and the withdrawal of drugs from the market (Segall and Barber, 2014). GF can cause transient mild-to-moderate elevation of serum transaminase levels in the treatment of superficial fungal skin infections, such as tinea capitis and tinea pedis, and a few reports have shown that it is associated with clinically significant acute drug-induced liver injury. However, the cause of obvious hepatotoxicity in clinical practice is still unclear. It is only mentioned that it is mainly widely metabolized by the liver and may lead to the production of toxic intermediates (Assenov et al., 2008). Han et al. found that after the oral administration of GF, GF was deposited on the epidermis through blood circulation and in the liver, muscle, and adipose tissue, resulting in pruritus, erythema, purpura, gastrointestinal discomfort, abdominal discharge, vomiting, etc. GF can cause an increase in the content of porphyrin in the blood, which can cause adverse reactions such as photoallergic dermatitis. In animal experiments, GF can affect the filamentous division of cells. Due to the increase in porphyrin content, it is decomposed and deposited on the bile duct wall by bile in the bile duct, causing necrosis and enlargement, causing toxic hepatitis. In addition, GF can also promote the carcinogenic effect of methylcholanthrene on the skin (Han, 1974). Tang et al. established a liver toxicity-prediction model using a network toxicology analysis method to predict the occurrence of liver toxicity that GF may induce, but it lacked experimental verification (Tang and Zhang, 2022). Clinical data showed that a female patient developed erythema and itching at the exposed part of the body after 1 week of oral griseofulvin tablets and was finally diagnosed as GF-induced photosensitive drug eruption. No abnormalities were found in the blood routine, urine routine, liver function, renal function, or chest X-ray after laboratory examination (Chen et al., 2020). No adverse reactions were found in the treatment of tinea capitis in children with GF (Kassem et al., 2023).

In summary, GF has certain toxicity and side effects when used in large quantities, so it should be used reasonably to prevent abuse and avoid adverse reactions. If GF is used, it should be avoided being combined with colchicine, barbiturate, phenytoin sodium, and other drugs that increase porphyrins in the blood so as not to increase toxicity; for long-term users, the white blood cell count, liver function, and urine and fecal porphyrin should be examined regularly during treatment (Han, 1974). In recent years, the research on GF has gradually reduced, which may be related to the serious side effects caused by its extensive use. Previous animal studies have shown that GF may cause hepatotoxicity and carcinogenicity (Epstein et al., 1967; Chlumská and Janousek, 1981; Rustia and Shubik, 1978). However, to date, there is not enough human research data to rule out human risks. Therefore, more animal models and clinical trials are needed to systematically evaluate its toxicity.

## 5 Conclusion and foresight

As a natural compound, the antitumor mechanism of GF mainly includes 1) inhibiting the proliferation and growth of tumor cells; 2) blocking the tumor cell cycle; 3) inhibiting tumor cell migration and invasion; 4) intervention of programmed cell death of tumor cells; and 5) the synergistic sensitizing effect of chemoradiotherapy. Studies have shown that GF has significant antitumor activity against various tumors, such as cervical cancer, breast cancer, non-small-cell lung cancer, colorectal cancer, and adrenocortical cancer. Its mechanism of action includes interfering with the cell cycle, inducing apoptosis, regulating Wnt/β-catenin, NF-κB, cGAS-STING, and other signaling pathways, and affecting the expression of p53, Bcl-2, Bax, cytochrome C, and other proteins (Ho et al., 2001; Rathinasamy et al., 2010; Bramann et al., 2013; Panda et al., 2005).

Transcriptional regulatory protein NF-kB is a key regulator of cell proliferation, differentiation, inflammation, apoptosis, and tumors. NF-kB can induce cell proliferation and promote tumor formation by activating proto-oncogenes such as C-myc and cyclin D1 (Yu et al., 2020; Pahl, 1999; Miller et al., 1999). It was proved for the first time that GF could induce G2/M phase arrest and apoptosis of HL-60 cells through the NF-kB pathway (Uen et al., 2007). The abnormal activation of the Wnt signaling pathway is closely related to the occurrence and development of tumors (He et al., 2021). Studies have found that GF can inhibit the Wnt/β-catenin signaling pathway in myeloma cell lines and induce apoptosis (Schmeel et al., 2017). In breast cancer MCF-7 cells, the IC_50_ value of GF was 17 μM. In multiple myeloma KMS 18 cells, the IC_50_ value of GF was 9 μM; for lymphoma SU-DHL-4 cells and small cell lung cancer NCI-H446 cells, the IC_50_ values of GF were 22 μM and 24.58 ± 1.32 μM; respectively. When acting on oral squamous cell carcinoma SCC114 cells, the IC_50_ value of GF was 6.4 μM (Rathinasamy et al., 2010; Schmeel et al., 2017; Shi, 2012; Liéby-Muller et al., 2015). GF can inhibit the proliferation, migration, and invasion of tumor cells, induce apoptosis, and inhibit tumor growth. In addition, GF can be combined with radiotherapy or drugs to obtain synergistic effects. GF combined with radiotherapy can make the tumor sensitive *in vivo*, and the effect of inhibiting tumor growth is ideal. The combination of GF and nocodazole can inhibit the growth cycle of cancer cells and the growth of tumors in immunodeficient mice. The combination of GF and vincristine can enhance the therapeutic effect on cancer cells (Ho et al., 2001; Rathinasamy et al., 2010). In order to improve the antitumor activity of GF, many studies have prepared a series of new GF derivatives with good antitumor activity by the structural modification of GF. It was found that when 2′-deoxy-2′-propoxygriseofulvin acted on SCC114 cells, its potency was about 10 times stronger than that of GF itself (Rebacz et al., 2007). When GF-15 acted on SCC114 cells *in vitro*, its potency was 27 times higher than that of GF itself (Liéby-Muller et al., 2015).

Current toxicity studies of GF mainly focus on hepatotoxicity and carcinogenicity. We note that the current literature on GF is limited and outdated and lacks convincing evidence. Therefore, we propose to further explore the pharmacological activity and toxicity of GF and study the pharmacology and toxicology of GF *in vivo* and *in vitro*. Only in this way can we provide a better basis for clinical application.

While summarizing it, it also puts forward its own views on several issues. For example, 1) the research on GF mainly stays in basic experiments, and there are few studies in clinical trials; 2) at present, the research on the mechanism of action of GF mainly focuses on a single signaling pathway or related targets. In view of the complexity and correlation of tumors, it is recommended to carry out multi-channel and multi-target mechanism research and exploration, such as the use of chemical biology methods to design and prepare GF molecular biological probes to discover and verify the exact drug targets of GF, which will fundamentally explain the pharmacological mechanism of GF and point out the direction for further modification of drug molecules; 3) the solubility and bioavailability of GF can be improved by glycosylation or preparation of nanomedicine so as to improve the druggability of GF; 4) at present, the pharmacological research of GF is mostly limited to *in vitro* experiments, and there are few studies at the animal level. To make the research more convincing, reasonable experiments should be carried out *in vitro* and *in vivo*; and 5) the research of GF mainly focuses on solid tumors such as colorectal cancer, breast cancer, cervical cancer, and lung cancer, and there are few studies on hematological tumors.

Pharmacological studies have shown that GF has antifungal, antitumor, antiviral, anti-inflammatory, and other effects. However, the pharmacological effects of GF are not deep enough, and the mechanism of action is not very clear, which needs further research and development. The antitumor effect and combination therapy can be the focus of future research. In addition, GF also has problems such as low bioavailability and limited distribution *in vivo*. Future research should focus on the development of GF composites or GF-loaded nanomaterials to improve their bioavailability.
